# Rare Case of Renal Cell Carcinoma with Mandibular Swelling as Primary Presentation

**DOI:** 10.1155/2013/806192

**Published:** 2013-06-25

**Authors:** Aleena Jallu, Manzoor Latoo, Rafiq Pampori

**Affiliations:** Department of Otolaryngology, Head and Neck Surgery, Government Medical College, Srinagar, Jammu and Kashmir, India

## Abstract

*Introduction.* Renal cell carcinoma accounts for approximately 3% of adult malignancies and 90–95% of neoplasms arising from the kidney. This disease is characterized by a lack of early warning signs, diverse clinical manifestations, and resistance to radiation and chemotherapy. Approximately one-third of patients with renal cell carcinoma have metastatic disease at initial presentation. Fifteen percent of patients with renal cell carcinoma are said to present with metastases in the head and neck region. Most of the metastases from RCC to the head and neck involve the thyroid gland. The head and neck are unusual sites for metastases, but skin, skeletal muscle, thyroid gland, nasal cavity and paranasal sinus metastases have been reported. *Case Report.* The following report describes a rare case where the patient presented with mandibular swelling of short duration as the primary complaint without any symptom or sign pertaining to urinary tract and was found to have renal cell carcinoma on further workup. *Conclusion.* Metastatic renal cell carcinoma is a diagnostic dilemma especially when there is no pointer historically towards renal cell carcinoma as was in our case. An unusual vascular osteolytic lesion in head and neck in a middle-aged person should be dealt with high index of suspicion with renal cell carcinoma at the back of the mind.

## 1. Introduction

Renal cell carcinoma is a kidney cancer that originates in the lining of the proximal convoluted tubule, the very small tubes in the kidney that filter the blood and remove waste products.

The classic triad of hematuria, flank pain, and an abdominal mass occurs only in 10–15% of cases, and is generally indicative of more advanced disease. Today, the majority of renal tumors are asymptomatic and are detected incidentally on imaging, usually for an unrelated cause.

Renal cell carcinoma is well known for its potential to metastasize to nearly every organ system in the body. The tumor is highly vascular and thought to metastasize via both hematogenous (via the Batson's plexus) and lymphatic routes [1]. The most common sites for metastasis are the lung, bone, adrenal, liver, brain, and the contralateral kidney [2]. Though not as frequent, metastatic renal cell carcinoma to the head and neck has been identified in the thyroid, salivary glands, skull base, sinuses, pharynx, tonsils, tongue, lip and skin [3]. Renal cell carcinoma is the third most frequent neoplasm to metastasize to the head and neck region preceded only by breast and lung cancer. Only in 1% of patients with advanced renal cell carcinoma metastases are limited exclusively to head and neck [4].

Metastasis is common in patients with a background of treated renal tumors, therefore determining the possibility of oral metastases is appropriate in such patients. The diagnosis of these metastases becomes a challenge though when there is no history of previous renal alterations [5, 6] and histopathologically, it is often confused with other neoplasia [5, 7]. It is in this type of patients that the diagnosis of carcinoma is achieved by evaluating for the metastasis. The present case represents one such incidence where the patient's only complaint was a rapidly progressing swelling in the left mandibular region and had no other systemic, abdominal or genitourinary complaints and was eventually found to have renal cell carcinoma on further evaluation.

## 2. Case Report

**Figure 1:**
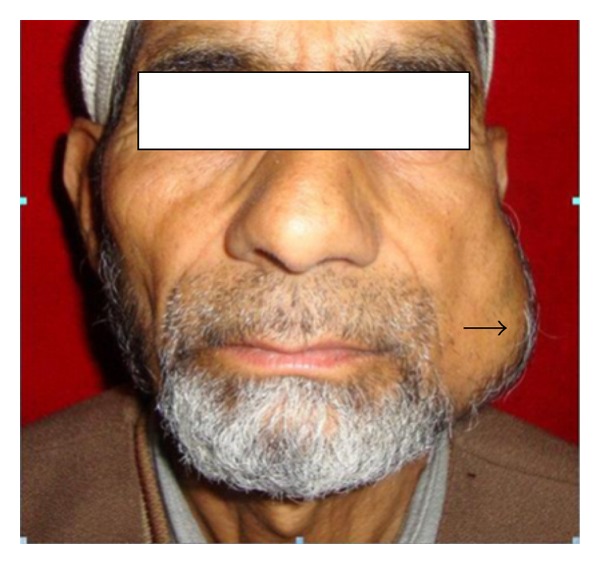
Clinical picture of the patient with one-month history of swelling in left mandibular region.

A 68-year-old male, formerly a smoker, presented with one-month history of rapidly progressing swelling in the left mandibular region (Figure 1). There was no other local or systemic complaint. Physical examination revealed a 5 × 5 cm firm, fixed, immobile, mildly tender swelling in region of left mandibular ramus. A firm, 1 × 2 cm, lobular, tender swelling was also noted in the region of left retromolar trigone. There was no other relevant finding on specific ENT and general examination. 

**Figure 2:**
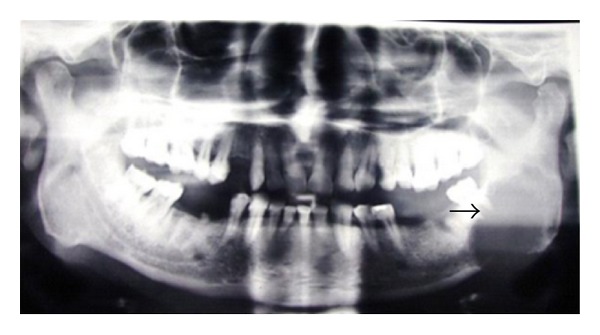
OPG showing osteolytic lesion involving left angle and ramus of mandible.

**Figure 3:**
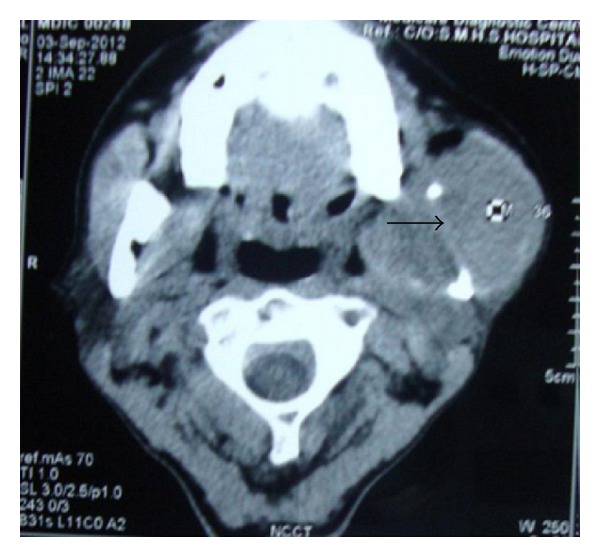
CT parotid region depicting osteolytic lesion in ramus and posterior body of mandible on left side.

 FNAC of the swelling was done and reported as myoepithelioma. Meanwhile OPG and CECT parotid regions were ordered. OPG showed an osteolytic lesion involving left ramus of mandible (Figure 2). CT scan of the parotid region revealed an osteolytic lesion involving inferior middle 3rd of left ramus of the mandible and posterior body of the mandible corresponding to the 3rd molar (Figure 3). There was erosion and effacement of the adjacent medial, lateral and inferior cortices of the ramus. The lesion involved left alveolar canal. Large soft tissue components extending medially and laterally involving the superficial and deep masticatory spaces were noted. Displacement of left masseter and medial pterygoid muscles was present. Doppler ultrasound of the neck revealed a well-defined soft issue mass lesion in relation to submandibular region with extensive central and peripheral vascularity arising predominantly from external carotid artery.

In view of the rapidly progressing vascular osteolytic lesion arising from within the mandible with normal panendoscopic examination of head and neck, we proceeded for metastatic workup, and FNAC report was put to question as myoepithelioma is a benign lesion both clinically as well as radiologically.

**Figure 4:**
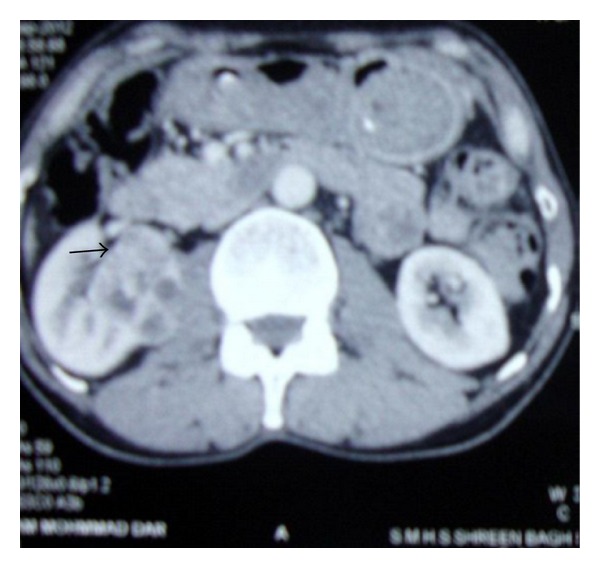
CECT abdomen, axial cuts showing well circumscribed mass arising from right kidney with stranding of perinephric fat.

**Figure 5:**
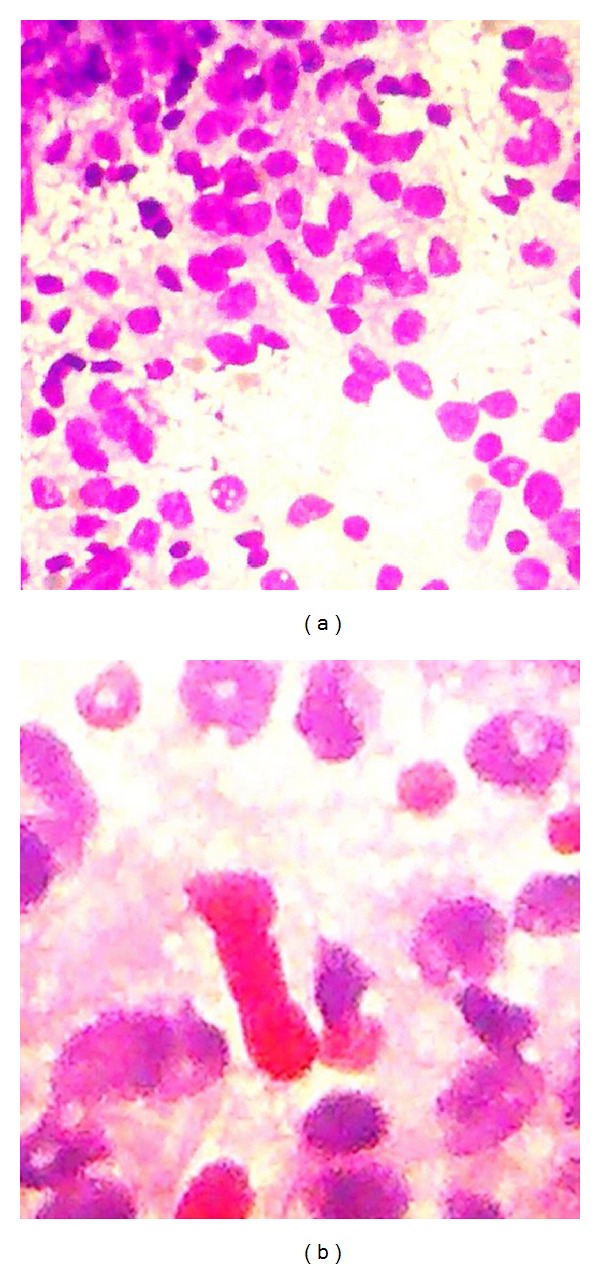
Histologic features of renal cell carcinoma; epithelial cellular network shown with clear cytoplasm and hyperchromatic nuclei surrounded by a rich vascular network.

Chest radiogram and CECT brain revealed normal scans. USG abdomen was done that showed a well-defined heterogenous mass (43 × 34 mm) in the right renal pelvis following which CECT abdomen was ordered that defined a well circumscribed mass in right kidney measuring 3.7 × 4.6 cm with stranding of perinephric fat and multiple B/L Bosniak type 1 renal cysts (Figure 4). With these radiological findings, the impression of renal cell carcinoma with mandibular metastasis was made, and the FNA slides from mandibular swelling were sent for review examination and reported as deposits of renal cell carcinoma (Figure 5). The patient was referred to the department of onco surgery for further management where right nephrectomy was done and the histopathological examination of the nephrectomy specimen was reported as renal cell carcinoma.

Postoperative PET scan of the patient was done to rule out other possible metastasis in the body which may have been missed by pre-op radiological workup. However, PET scan revealed that only one lesion confines to mandible. The sole metastasis in the mandible being highly vascular had by then grown considerably with erosion of retromolar trigone plus tonsil, and the fungating growth with teeth on it was seen hanging in the oral cavity and oropharynx. Patient had by now started bleeding from the oral lesion. Since the volume and the bulk of the tumor were quite, huge target therapy could not be contemplated, and the patient was subjected to gadolinium enhanced MRI of the oral cavity and neck with the intension of surgery of sole metastasis in the form of wide excision plus hemimandibulectomy and reconstruction with pectoralis major flap. However MRI revealed extensive destructive lesion with tumor tissue abutting major vessels of head and neck including internal carotid artery, and hence patient was subjected to palliative chemoradiation.

## 3. Discussion

Renal cell carcinoma is a malignant pathology of difficult and many times tardy diagnosis [5, 8–10]. The clinical behaviour of metastatic renal cell carcinoma is unpredictable, and the signs and symptoms depend on the affected organ and the presence of local invasion. Metastatic deposits from renal carcinoma are vascular in nature and often bleed.

The rich venous anastomosis with the prevertebral, vertebral, and epidural system supports a pathway for the tumor to spread. Veins, which are valveless, offer an easy way for tumor emboli to spread with less resistance. Increase in intra-abdominal or intrathoracic pressure causes a retrograde flow from the venous channels back through the prevertebral and vertebral venous plexus. In this way, renal carcinoma apparently can travel from the kidney, bypass the pulmonary capillary filtration, and metastasize in the head and neck. If there is no evidence of lung or liver disease, it has been postulated that tumor cells can migrate through Batson's venous plexus or the lymphatics via the thoracic duct. This clearly defines the pathway for right-sided renal cell carcinoma going to left mandible in our case.

Clear cell carcinoma is the most common histological variant of renal cell carcinoma. The clear cells are rounded or polygonal with abundant cytoplasm, which contains cholesterol, cholesterol esters, phospholipids, and glycogen. These are dissolved during routine histological processing, creating a clear cytoplasm surrounded by a distinct cell membrane. Histopathological examination commonly shows a network of small, thin-walled blood vessels interlacing between the tumour cells.

Clear cell renal cell carcinoma is a challenging diagnosis for the pathologist, as the differential diagnosis includes benign tumors, such as oncocytic hyperplasia, oncocytoma, *myoepithelioma*, pleomorphic adenoma, and sebaceous adenoma [11]. This explains the diagnostic discrepancy in the cytologic examination before and after.

There are reports of patients with a history of renal cell carcinoma who presented metastases years later [12]. Some of these metastatic sites in head and neck that have been reported in the literature include parotid [13, 14], paranasal sinuses [15], nasopharynx [15], oral cavity [16], and lip [17]. Within the literature, investigators determined an interval of 5 to 36 months between the appearance of the primary tumor and the metastasis. The problem is those patients who do not have a history of malignant nephropathy [5, 6] in which the clinics becomes relevant [7, 12, 18], given that its expression can be multiple and unspecific; a complicated epistaxis [12], lingual tumors [6], neurological alterations [19], paresthesias [18], strokes [8], visual alterations [8], signs of hypopituitarism [8] or impotence [8].

The present case is reported for its uniqueness as mandibular swelling being the initial presentation of renal cell carcinoma. In the present case the systemic history and examination were noncontributory. It was only after the USG abdomen and Doppler USG of the neck, a suspicion of metastatic lesion from renal cell carcinoma to mandible that arose for which pathologist was asked to review the slides. Metastatic tumors to the jaws are relatively uncommon and involve the mandible more frequently than the maxilla. When they occur, the primary tumor is most likely to be an adenocarcinoma from the breast, lung, or kidney. A pulsatile mandibular lesion, found not to be an arteriovenous fistula, suggests a metastatic renal carcinoma until it is disproved [20]. 

Although the systemic spread of a metastatic tumour excludes a rationale for local treatment, for example, metastasectomy, this is actually a feasible option for RCC [21, 22]. In fact, despite not being supported by a high level of evidence, over time, this approach has obtained wide consensus both due to the fact that in some cases it allows a prolonged survival and clinical regression of the disease and because there is a lack of more effective therapeutic alternatives. Even targeted therapies that have recently been introduced into clinical practice, while leading to a significant increase in survival compared with previous therapies [21, 23–25], have not replaced the use of metastasectomy due to their limited ability for complete regression of the disease and the need to extend treatment and the related side effects, for an unlimited period [21, 26–28]. There are published reports on the results of metastasectomy in the most common sites of RCC metastasis (lung, bone, liver, brain, and adrenal) [29, 30]. However, this tumour can, even though unusual, spread to any organ, and only single case reports describe metastasectomies in “atypical” sites.

Our search of, literature could find only one or two cases of renal cell carcinoma presenting as mandibular swell [31, 32] but these patients had symptoms pertaining to urinary tract or abdomen.

On account of this we feel that ours may be the first or among the first of such cases.
